# Reassessment of Postural Stimulation Testing as a Simple Tool to Identify a Subgroup of Patients With Unilateral Primary Aldosteronism

**DOI:** 10.1210/clinem/dgab611

**Published:** 2021-08-20

**Authors:** Carmina Teresa Fuss, Katharina Brohm, Martin Fassnacht, Matthias Kroiss, Stefanie Hahner

**Affiliations:** 1 Division of Endocrinology and Diabetes, Department of Internal Medicine I, University Hospital, University of Würzburg, Würzburg, Germany; 2 Central Laboratory, Core Unit Clinical Mass Spectrometry, University Hospital Würzburg, Würzburg, Germany; 3 LMU Klinikum, Department of Internal Medicine IV, Munich, Germany

**Keywords:** aldosterone-producing adenoma, bilateral hyperplasia, adrenal vein sampling

## Abstract

**Context:**

Adrenal vein sampling (AVS) represents the current diagnostic gold standard for differentiation between unilateral and bilateral primary aldosteronism (PA). Postural stimulation testing (PST) has been used to provide additional diagnostic information.

**Objective:**

This work aimed to evaluate the diagnostic utility of PST in the differential diagnosis of PA.

**Methods:**

This cohort study was conducted at a single tertiary reference center. We analyzed 106 PST performed between 2008 and 2020. Diagnosis of PA and cause of PA were determined according to the Endocrine Society Clinical Practice Guideline, taking into account results of saline infusion testing, AVS, preoperative imaging, and outcome after medical or surgical treatment. The suggested cutoffs for the diagnosis of unilateral PA were revisited and optimized for high specificity using receiver operating characteristics (ROC) analysis.

**Results:**

A total of 106 patients had confirmed PA (unilateral PA: n = 55, bilateral PA: n = 29, AVS unsuccessful/declined by patients: n = 22). Based on decreased aldosterone plasma concentration of 28% or more after 4 hours in the upright position, the PST showed a sensitivity of 36.4% at a specificity of 100% to identify unilateral disease (area under the curve [AUC] = 0.72; 95% CI, 0.62-0.83; *P* = .001). In patients with valid testing (drop of cortisol of 10% or more after 4 hours, n = 53) the sensitivity of PST rose to 51.4% at a specificity of 100% (AUC = 0.77; 95% CI, 0.65-0.90; *P* = .001).

**Conclusion:**

The high specificity of 100% for the detection of unilateral PA in patients with decreased aldosterone by at least 28% after 4 hours makes PST a simple, noninvasive contribution to subtype differentiation in PA.

Primary aldosteronism (PA) is the most common form of endocrine hypertension (1). Correct identification of unilateral vs bilateral PA caused by aldosterone-producing adenoma, APA, and bilateral micronodular hyperplasia, BAH, respectively, guides the clinical management. While unilateral disease can potentially be cured by surgery, patients with BAH require lifelong treatment with mineralocorticoid receptor antagonists (2). Even though novel, noninvasive approaches to differentiate between subtypes show promising results (3-9), adrenal vein sampling (AVS) still represents the current diagnostic reference method for differentiation between APA and BAH. The disadvantages of AVS include invasiveness, costs, radiation exposure, and a lack of standardization both of the procedure and the interpretation (10-12). Importantly, the AVS success rate is highly dependent on the experience of the performing radiologist (10, 13) and AVS results are sometimes inconclusive, mostly because of unsuccessful cannulation of the adrenal veins (11).

Postural stimulation testing (PST) for subtype differentiation in PA was first proposed in the 1970s (14). Patients with BAH show higher sensitivity to angiotensin II leading to increased aldosterone after postural stimulation. In line with previous studies (15, 16), in a recent comprehensive analysis of aldosterone response to different stimuli in PA, all patients with BAH (n = 11) showed an increase in aldosterone after 2 hours in the upright posture (17). On the contrary, some patients with APAs respond to changes in posture with a decrease in aldosterone (18-22). All published studies taken together have analyzed fewer than 350 patients, with conflicting results. Therefore, only a few centers still perform PST as part of the diagnostic workup in patients with PA and some see it as a mostly historic test.

The present study reevaluates the diagnostic utility of PST in the differential diagnosis of PA at a single center in a cohort of 160 consecutively tested patients.

## Materials and Methods

### Patient Cohort

In this retrospective study we reviewed 160 consecutive PST performed at the University Hospital Würzburg between 2008 and 2020 in patients who were referred for confirmatory testing for PA. Diagnosis of PA as well as subtype of PA (unilateral vs bilateral disease) was determined clinically according to the Endocrine Society Clinical Practice Guidelines (2), taking into account results of saline infusion testing, preoperative imaging, AVS, adrenal pathology, and outcome after surgical treatment.

### Postural Stimulation Testing

To perform confirmatory testing for PA, antihypertensive therapy had been discontinued or replaced by antihypertensive drugs that limit interference with the renin-angiotensin-aldosterone system (eg, α1-receptor-antagonists or calcium channel blockers). All patients underwent PST regardless of the results of saline infusion testing. PST was performed in a controlled inpatient setting. The first blood sample was taken in the morning between 8 and 9 am at the end of overnight bed rest, and a second one after 4 hours in an upright position. Aldosterone, cortisol, and renin concentrations were quantified in both samples.

### Measurement of Aldosterone, Cortisol, and Renin

Test results were derived from routine testing at the time of test performance. Until September 2014 serum aldosterone was determined by radioimmunoassay Coat-a-Count (Siemens) and later by the automated chemiluminescence immunoassay IDS-iSYS Aldosterone (Immuno Diagnostic Systems). Renin measurement was performed by radioimmunometric assay Renin 3rd Generation (Cisbio) until September 2014 and with the automated chemiluminescence immunoassay IDS-iSYS Direct Renin assay (Immuno Diagnostic Systems). Serum cortisol was analyzed with the chemiluminescent enzyme immunoassay IMMULITE 2000 Systems Analyzers (Siemens).

### Statistical Analysis

Of the 320 samples collected during PST, 11 samples showed aldosterone values below the lower limit of quantification (LLOQ, < 37 ng/L). In those cases, we used an aldosterone concentration based on the following formula for statistical analysis: c = LLOQ/√2 (random number between 0.75 and 1.5). Data are shown as mean ± SD, as median and interquartile range, or as absolute numbers and percentages, as appropriate. Mann-Whitney *U* tests for quantitative nonnormally distributed variables was used to compare patients with and without PA, and Wilcoxon test for paired samples. Receiver operating characteristics (ROC) curves were used to establish an optimized aldosterone threshold to distinguish between unilateral and bilateral disease. A *P* value less than .05 was considered statistically significant. Statistical analysis was performed using SPSS (v 25; IBM Corp) and MedCalc (v 19.3).

## Results

### Patient Cohort

A total of 106 patients with confirmed PA and 54 patients with essential hypertension (EH) in whom PA was excluded based on the results of saline infusion testing were included in the final analysis. The clinical characteristics of both groups are given in Table 1. Patients with PA showed higher body mass index, a higher rate of comorbidities, and a higher proportion of potassium supplementation. In patients with PA, subtype differentiation was possible in 79% of cases (n = 84). Fifty-five patients (65.5%) presented with unilateral and 29 (34.5%) with bilateral disease. Thirty-five patients with unilateral PA had sufficient follow-up (4 months-132 months) after adrenalectomy (surgically treated patients: n = 50). Of these, according to the Primary Aldosteronism Surgery Outcome treatment criteria (23), 13 had complete and 20 partial clinical success, success was absent in 2. Thirty-three patients showed complete and 2 patients partial biochemical success. Table 2 shows an overview of results of adrenal imaging (computed tomography [CT] or magnetic resonance imaging [MRI]) in all patients.

**Table 1.** Clinical characteristics of the entire sutdy cohort

	PA	EH	P
Age, y	52 ± 12	52 ± 15	.737
Male, No. (%)	69 (65%)	20 (37%)	.001
BMI	28.3 (25.9-32.2)	26.5 (23.8-29.7)	.043
Diabetes mellitus, No. (%)	14 (13%)	2 (4%)	.059
Coronary heart disease, No. (%)	4 (4%)	2 (4%)	.983
Stroke, No. (%)	6 (6%)	2 (4%)	.592
Renal insufficiency, No. (%)	10 (9%)	1 (2%)	.074
Sleep apnea syndrome, No. (%)	6 (6%)	3 (6%)	.484
Potassium, mmol/L	4.0 (3.4-4.3)	4.3 (4.0-4.5)	< .001
Sodium, mmol/L	142 (141-143)	141 (139-143)	< .001
GFR, mL/min	92 (78-103)	87 (77-96)	.382
Systolic BP, mm Hg	153 (142-165)	149 (138-158)	.118
Diastolic BP, mm Hg	90 (83-97)	83 (80-92)	.003

Data are given as mean ± SD or median and interquartile range.

Abbreviations: BMI, body mass index; BP, blood pressure; EH, essential hypertension (n = 54); GFR, glomerular filtration rate; PA, primary aldosteronism (n = 106).

**Table 2.** Results of adrenal imaging

	PA No. (%)	EH No. (%)
No adrenal pathology	31 (29)	8 (15)
Unilateral nodule	59 (56)	11 (20)
Bilateral nodules	7 (7)	1 (2)
No adrenal imaging	9 (9)	34 (63)

Results of adrenal imaging (computed tomography or magnetic resonance imaging) in patients with primary aldosteronism (PA, n = 106) and essential hypertension (EH, n = 54).

### Hormone Concentrations During Postural Stimulation Testing

**Figure 1.**
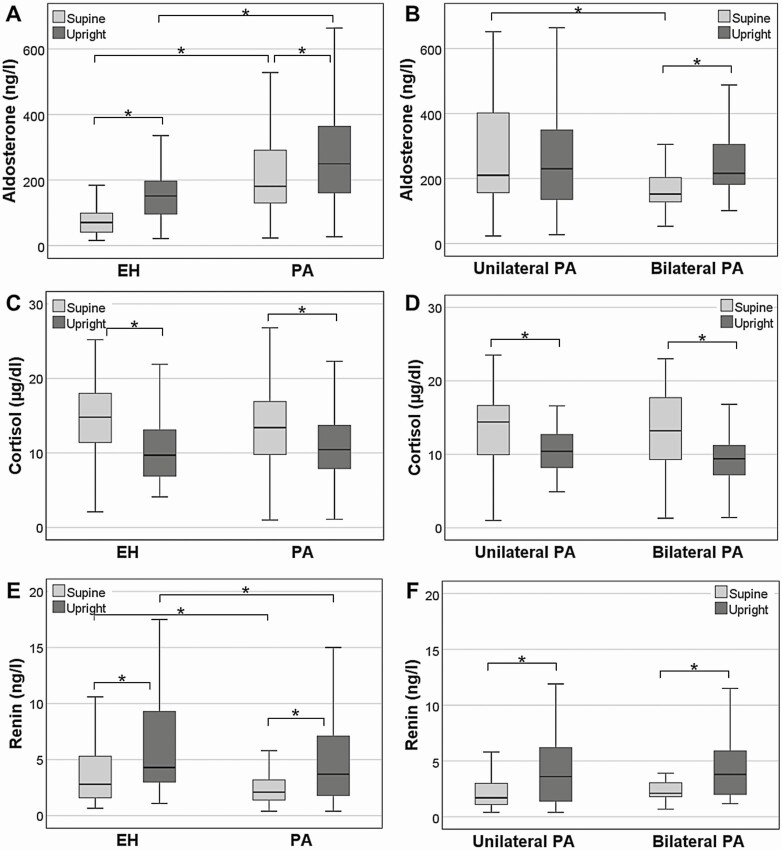
Differences in A and B, aldosterone; C and D, cortisol and renin; and E and F, levels during postural stimulation testing in patients with essential hypertension (EH, n = 54) and primary aldosteronism (PA, n = 106), as well as unilateral (n = 55) and bilateral (n = 29) primary aldosteronism. *P less than .05.

**Figure 2.**
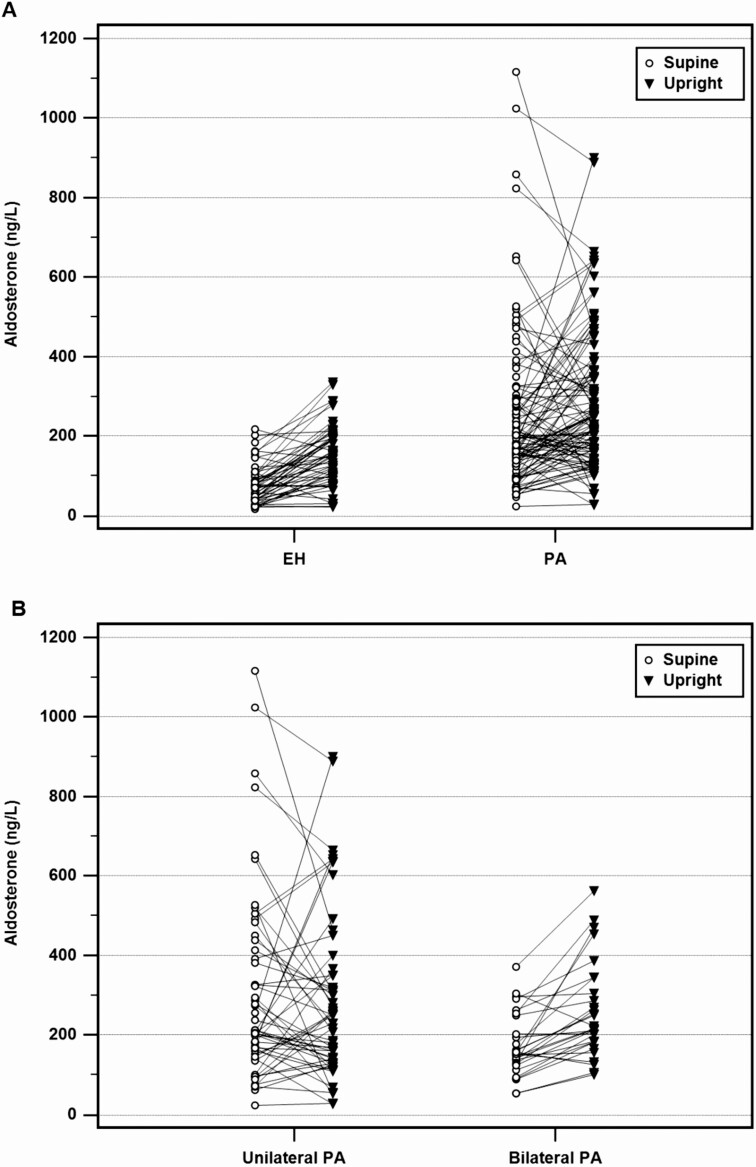
Aldosterone concentrations before and after postural stimulation in single patients with A, essential hypertension (EH, n = 54); A, primary aldosteronism (PA, n = 106); B, unilateral PA (n = 55); and B, bilateral PA (n = 29).

On average, patients with PA as well as EH showed a significant increase of renin, increase in aldosterone concentrations, and decrease of cortisol and after four hours in an upright position (Fig. 1, Table 3). Plasma aldosterone concentration did not differ significantly between the supine and upright position in unilateral PA, but exhibited a significant increase in patients with BAH after 4 hours (see Fig. 1B, Table 3). Patients with unilateral PA presented with higher aldosterone levels at baseline. Aldosterone concentration after 4 hours increased in 83% of patients with EH, 93% of patients with bilateral PA, and only 40% of patients with unilateral PA. Aldosterone dynamics in single patients with EH, PA, unilateral and bilateral PA are depicted in Fig. 2.

**Table 3.** Concentrations of aldosterone, cortisol and renin during postural stimulation testing in the study cohort

	PA	EH	P	Unilateral PA	Bilateral PA	P
Aldosterone supine, ng/L	183 (137-295)	69 (41-94)	< .001	210 (157-426)	154 (129-238)	.004
Aldosterone upright, ng/L	252 (166-366)	154 (96-203)	< .001	249 (136-358)	220 (183-334)	.821
Cortisol supine, µg/dL	13.6 (10.3-17.3)	14.0 (11.4-18.2)	.094	14.4 (9.8-16.9)	13.2 (9.5-17.7)	.611
Cortisol upright, µg/dL	10.6 (8.1-13.7)	9.1 (6.9-13.0)	.465	10.4 (8.2-12.7)	9.7 (7.2-11.7)	.364
Renin supine, ng/L	2.1 (1.4-3.2)	2.8 (1.6-5.5)	.029	1.7 (1.1-3.1)	2.1 (1.8-3.1)	.216
Renin upright, ng/L	3.7 (1.8-7.2)	4.3 (3.0-12.9)	.041	3.6 (1.4-6.4)	3.8 (2.0-6.0)	.491

Concentrations of aldosterone, cortisol, and renin in supine and upright conditions for patients with primary aldosteronism (PA, n = 106), essential hypertension (EH, n = 54), unilateral primary aldosteronism (n = 55), and bilateral primary aldosteronism (n = 29). Data are shown as median and interquartile range.

### Diagnostic Accuracy of Postural Stimulation Testing

**Figure 3.**
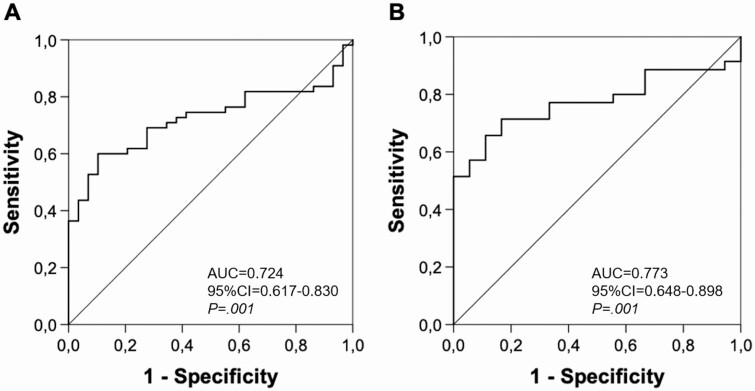
Receiver-operating-characteristics (ROC) curve for aldosterone after 4 hours in the upright position for prediction of unilateral primary aldosteronism (PA). A, ROC curve for whole PA-cohort (unilateral PA: n = 55, bilateral PA: n = 29). B, ROC curve for patients with cortisol drop of 10% or more after 4 hours in the upright position (unilateral PA: n = 35, bilateral PA: n = 18). AUC, area under the curve.

By using the proposed cutoff of a drop in aldosterone of 30% or more after postural stimulation (19-21), PST showed a specificity of 100% at a sensitivity of 34.5% for prediction of unilateral PA. ROC analysis revealed a reduction of aldosterone of 28% or more to still provide a specificity of 100% at a slightly improved sensitivity of 36.4% and positive predictive value of 100% (area under the curve = 0.724; 95% CI, 0.617-0.830; *P* = .001, Fig. 3A). In our cohort, 20 out of 55 patients with unilateral PA showed an aldosterone reduction of 28% or more (Table 4).

**Table 4.** Sensitivity and specificity of different aldosterone cutoffs after postural stimulation for detection of unilateral disease

Aldosterone change %	Sensitivity % (95% CI)	Specificity % (95% CI)	+LR (95% CI)	–LR (95% CI)	TP No.	FP No.	TN No.	FN No.	PPV %	NPV %
≤ –30	34.6 (22.2-48.6)	100.0 (88.1-100.0)	–	0.7 (0.5-0.8)	19	0	29	36	100.0	44.6
≤ –28	36.4 (23.8-50.4)	100.0 (88.1-100.0)	–	0.6 (0.5-0.8)	20	0	29	35	100.0	45.3
≤ –27	36.4 (23.8-50.4)	96.6 (82.2-99.9)	10.6 (1.5-74.7)	0.7 (0.5-0.8)	20	0	29	35	100.0	45.3
≤ –25	38.2 (25.4-52.3)	96.6 (82.2-99.9)	11.1 (1.6-78.2)	0.6 (0.5-0.8)	21	1	28	34	95.5	46.8
≤ –24	40.0 (27.0-54.1)	96.6 (82.2-99.9)	11.6 (1.6-81.8)	0.6 (0.5-0.8)	22	1	28	33	95.7	45.9
≤ –22	41.8 (28.7-55.9)	96.6 (82.2-99.9)	12.1 (1.7-85.3)	0.6 (0.5-0.8)	23	1	28	32	95.8	46.7
≤ –20	45.5 (32.0-59.4)	93.1 (77.2-99.2)	6.6 (1.7-25.9)	0.6 (0.5-0.8)	25	2	27	30	95.6	47.4
≤ –16	50.9 (37.1-64.6)	93.1 (77.2-99.2)	7.4 (1.9-28.8)	0.5 (0.4-0.7)	28	2	27	27	93.3	50.0
≤ –13	52.7 (38.8-66.3)	93.1 (77.2-99.2)	7.7 (2.0-29.8)	0.5 (0.4-0.7)	29	3	26	26	90.6	50.0
≤ –9	54.6 (40.6-68.0)	89.7 (72.6-97.8)	5.3 (1.8-15.8)	0.5 (0.4-0.7)	30	3	26	25	90.9	51.0
≤ –7	56.3 (42.3-69.7)	89.7 (72.6-97.8)	5.5 (1.8-16.3)	0.5 (0.4-0.7)	31	3	26	24	91.2	52.0
≤ –4	60.0 (45.9-73.0)	89.7 (72.6-97.8)	5.8 (1.9-17.3)	0.5 (0.3-0.6)	32	3	26	23	91.4	53.1
≤ 0	60.0 (45.9-73.0)	86.2 (68.3-96.1)	4.4 (1.7-11.1)	0.5 (0.3-0.7)	33	4	25	22	89.2	53.2

Sensitivity and specificity of different aldosterone cutoffs after postural stimulation for detection of unilateral disease in the whole cohort (n = 84).

Abbreviations: Aldosterone change, change in aldosterone after postural stimulation (%); FN, false negative; FP, false positive; +LR, positive likelihood ratio; –LR, negative likelihood ratio; NPV; negative predictive value; PPV, positive predictive value; TN, true negative; TP, true positives.

Restricting the analysis to patients with PA with a drop in cortisol of 10% or more after 4 hours in the upright position (n = 53), a 28% or more reduction in aldosterone after postural stimulation further increased specificity to 51.4% (area under the curve = 0.773; 95% CI, 0.648-0.898; *P* = .001; Fig. 3B; Supplementary Table 1) (24).

Eight patients underwent adrenalectomy without prior AVS. Five of those showed a reduction in aldosterone of ≥28%, the remaining three of at least 19%. In 5/8 cases follow-up data was available. All five patients benefited from surgical intervention.

### Combined Analysis of Postural Stimulation Testing and Imaging

Of the 106 patients with confirmed PA, 59 had a unilateral nodule on CT or MRI scans. Of these patients, 18 had a PST result indicating APA (defined by drop in aldosterone of ≥ 28%).

## Discussion

Correct subtype differentiation in patients with PA remains challenging. To limit AVS to a minimum, given its numerous drawbacks, we aimed to assess the utility of PST in a cohort of consecutively tested patients with PA.

In our study, most patients with EH showed a physiological response to postural stimulation with an angiotensin II–mediated increase in aldosterone. The same reaction to activation of the renin-angiotensin-aldosterone system was observed in bilateral PA. In contrast, only 40% of patients with unilateral PA had an increase in aldosterone after 4 hours in the upright position. Even more important, in 20 of 55 patients with unilateral PA a decrease of aldosterone of at least 28% was detectable, leading to a sensitivity of 36.4% at a specificity of 100%.

The concept of the PST relies on the observation that aldosterone secretion can be stimulated by adrenocorticotropin in addition to renin (25, 26). The test uses the circadian rhythm with higher adrenocorticotropin in the morning in addition to postural stimulation, hence the test principle is realized only when a drop of cortisol is observed during the test.

Reported diagnostic performance of PST differs in the literature. Espiner et al (27) evaluated 35 patients with unilateral PA by PST. A total of 65% showed a decrease in aldosterone, leading to a high specificity and positive predictive value of 100% in their analysis, which was however interpreted independently of cortisol dynamics during the test. A series of 50 PST with blood sampling after 1 and 4 hours in the upright position suggested poor diagnostic accuracy for differentiation between APA and BAH (for APA: sensitivity = 56%, specificity = 75% after 4 hours) using a decrease in aldosterone of 30% or more as suggestive for APA and an increase of 30% or more as evidence for BAH (21).

In an analysis of 146 PA patients, Fontes et al (20) suggested that any patient with PA, and an increase of aldosterone by up to 30% in the PST and a detectable tumor on imaging, could potentially benefit from surgery. When applying these criteria to our cohort, we would have 35 patients with ‘positive PST,’ but this would have led to a misclassification of 5 patients as unilateral in whom bilateral PA would have been suggested by PST alone.

Indeed, when we limit our analysis to patients with a decrease of cortisol during the 4 hours of PST of at least 10%, the sensitivity proved to be higher (51.4%).

In the patients with adrenal nodule on CT or MRI scans, PST was able to correctly classify one-third of patients.

### Limitations

Given the retrospective nature of this study over a longer period, changes in clinical management, AVS procedure, and AVS experience are inevitable. Aldosterone concentration was measured using 2 different assays, which may limit the comparability of cases before and after 2014. However, this should have had a minor effect on the assessment of percentage variation. The fairly high prevalence of unilateral disease in our study may point toward selection bias regarding the study setting and more florid forms of PA in our tertiary care center. Subtype differentiation was not performed in all cases of PA (mostly according to patient preference), and not all patients who underwent adrenalectomy had sufficient follow-up data for outcome assessment.

In conclusion, PST is certainly not a substitution for AVS. However, it is a relatively simple, cheap, and easy to perform a test that might improve PA subtype diagnosis, especially in centers with limited experience with AVS or limited access to AVS. Current guidelines suggest performing adrenalectomy in patients younger than 35 years with confirmed PA, spontaneous hypokalemia, and evidence of an adrenal adenoma on imaging. In our view, PST may render AVS dispensable in additional patients of younger age with a high probability of unilateral disease who do not meet these strict criteria.

## Data Availability

The data sets generated during and/or analyzed during the present study are not publicly available but are available from the corresponding author on reasonable request.

## Abbreviations

APA: aldosterone-producing adenoma
AVS: adrenal vein sampling
BAH: bilateral micronodular hyperplasia
CT: computed tomography
EH: essential hypertension
LLOQ: lower limit of quantification
MRI: magnetic resonance imaging
PA: primary aldosteronism
PST: postural stimulation testing
ROC: receiver operating characteristic

## Additional Information

**Disclosures:** The authors have nothing to disclose.
